# Foliar application of the leaf-colonizing yeast *Pseudozyma churashimaensis* elicits systemic defense of pepper against bacterial and viral pathogens

**DOI:** 10.1038/srep39432

**Published:** 2017-01-10

**Authors:** Gahyung Lee, Sang-Heon Lee, Kyung Mo Kim, Choong-Min Ryu

**Affiliations:** 1 Molecular Phytobacteriology Laboratory, Superbacteria Research Center, KRIBB, Daejeon 305-806, South Korea; 2 Microbial Resource Center, KRIBB, Jeongeup 56212, South Korea; 3 Department of Bioinformatics, University of Science and Technology (UST), Daejeon 34141, South Korea; 4 Biosystems and Bioengineering Program, School of Science, University of Science and Technology (UST), Daejeon 34113, South Korea

## Abstract

Yeast associates with many plant parts including the phyllosphere, where it is subject to harsh environmental conditions. Few studies have reported on biological control of foliar pathogens by yeast. Here, we newly isolated leaf-colonizing yeasts from leaves of field-grown pepper plants in a major pepper production area of South Korea. The yeast was isolated using semi-selective medium supplemented with rifampicin to inhibit bacterial growth and its disease control capacity against *Xanthomonas axonopodis* infection of pepper plants in the greenhouse was evaluated. Of 838 isolated yeasts, foliar spray of *Pseudozyma churashimaensis* strain RGJ1 at 10^8^ cfu/mL conferred significant protection against *X. axonopodis* and unexpectedly against *Cucumber mosaic virus, Pepper mottle virus, Pepper mild mottle virus,* and *Broad bean wilt virus* under field conditions. Direct antagonism between strain RGJ1 and *X. axonopodis* was not detected from co-culture assays, suggesting that disease is suppressed via induced resistance. Additional molecular analysis of the induced resistance marker genes *Capsicum annuum Pathogenesis-Related (CaPR) 4* and *CaPR5* indicated that strain RGJ1 elicited plant defense priming. To our knowledge, this study is the first report of plant protection against bacterial and viral pathogens mediated by a leaf-colonizing yeast and has potential for effective disease management in the field.

Foliar pathogens cause global economic and yield losses of major crop plants^1^. Fungi are the predominant foliar pathogen that contributes to these losses^1^. Essentially, all of the important fungal pathogens can be controlled by the use of agrochemicals^2^. Although bacterial and viral pathogens affect relatively fewer crops and cause less pervasive losses, there is a lack of effective control methods for these pathogens^2^,^3^. In agriculture, antibiotics are used to control bacterial pathogens on certain trees, but these have severely adverse collateral effects such as the spread of antibiotic resistance among bacteria and secondary delivery of antibiotics to animals and humans^4^,^5^. Virus-induced plant diseases are becoming a critical issue of concern for farmers^6^; however, there are currently no control methods against viral diseases except for engineering or breeding virus-resistant plants^7^,^8^.

Chemical and biological agents can be used to enhance plant basal immunity. This method is referred to as “induced resistance”^9^,^10^. These studies provide insights into possible management solutions for challenging plant diseases such as those caused by bacterial and viral pathogens. *Tobacco mosaic virus* (TMV) can induce systemic acquired resistance (SAR) in plants to potentiate resistance responses to *Tobacco necrosis virus, Turnip mosaic virus*, and *Tobacco*/*Tomato ringspot viruses*^11^. Plant pathogens and beneficial microbes can induce plant resistance, which has beneficial effects for plant growth crop yield^9^. Pathogen application to elicit plant resistance responses in the field is difficult, and chemical inducers have significantly adverse side effects for plant growth and crop yield^11^,^12^,^13^. Therefore, the use of biological agents to induce plant resistance has become increasingly attractive. Biological agents that induce plant resistance to pathogens include bacteria and fungi that naturally associate with plants.

Among biological agents, plant growth-promoting rhizobacteria (PGPR) have been used to reduce disease symptoms and pathogen titers caused by bacteria and viruses, and developed into biological products^14^,^15^,^16^. For example, a *Bacillus pumilus* INR7 endophyte was commercialized as Yield Shield; seed treatment with Yield Shield successfully managed *Cucumber mosaic virus* (CMV) on pepper (*Capsicum annuum*), angular leaf spot caused by *Pseudomonas syringae* pv. *lachrymans* on cucumber, and bacterial wilt caused by *Erwinia tracheiphila* on cucumber during field trials^17^,^18^,^19^,^20^,^21^. Soil amendment with a commercially available bacterial bioproduct (BioYield containing *B. subtilis* GB03 and *B. amyloliquefaciens* IN737a) before pepper seedling transplantation protected against CMV in the greenhouse and reduced the incidence and severity of *Tomato mottle virus*, CMV, and *Ralstonia solanacearum* in the field^17^,^22^,^23^,^24^. The combination of strain *B. pumilus* INR7 and the chemical trigger benzothiadiazol has a synergistic effect in eliciting induced resistance against bacterial spot and stimulating defense gene expression in pepper plants compared with the effects of single bacterial or chemical treatments^21^.

Leaf-colonizing yeasts are widely used as biological control agents to protect against diverse foliar pathogens such as powdery mildew fungi, *Aspergillus flavus, Botrytis*, and *Ustilago maydis*^25^,^26^. *Pseudozyma flocculosa* is a basidiomycetous fungal yeast that has been extensively characterized as an effective control agent for powdery mildew fungi, which are ubiquitous phyllosphere pathogens of numerous field and greenhouse crops. *P. flocculosa* was first isolated as an antagonist of cucumber powdery mildew under different environmental conditions^27^. Subsequent work showed that it was equally effective against *Spharotheca pannosa* var. rosae and *Erysiphe graminis* f. sp. tritici, which are responsible for rose and wheat powdery mildew, respectively^28^,^29^. There is commercial interest to develop *P. flocculosa* as a biofungicide (based on *P. flocculosa* conidia)^30^. An early study reported that *P. flocculosa* does not penetrate plants but induces rapid plasmolysis of powdery mildew cells, which suggests that *P. flocculosa* secretes an antibiotic or other bioactive agent that affects powdery mildew cells^31^. Subsequent work showed that *P. flocculosa* culture filtrates produced the same effects (rapid cell plasmolysis) on powdery mildew fungi^32^. Molecular and biochemical analyses identified the antibiotic glycolipid flocculosin, and found that flocculosin production was strongly correlated with *cyp1* expression, which encodes a monooxygenase with a crucial role in fungal growth inhibition^33^. Local inoculation of *Pseudozyma aphidis* elicited induced resistance in *Arabidopsis* and reduced growth of the necrotrophic fungus *Botrytis cinerea* on local and systemic leaves^34^. Induced resistance was confirmed by gene expression priming analysis of *PR1* and *PDF1.2* in *Arabidopsis*^34^. The defense signaling for the *P. aphidis*-mediated induced resistance was elicited through pathways that were dependent on jasmonic acid (JA) and salicylic acid (SA) signaling but independent of ethylene signaling^35^,^36^. Pre-treatment spraying of tomato and cucumber plants with *Podosphaera xanthii* spores suppressed bacterial canker (caused by *Clavibacter michiganensis*) and powdery mildew (caused by *Podosphaera xanthii*). Further analysis revealed that extracellular metabolites from *P. aphidis* directly inhibited *C. michiganensis* growth and *P. xanthii* spore germination; therefore, this cannot be classified as induced resistance^35^,^36^.

The primary objectives of this study were to isolate and characterize leaf-colonizing yeasts with potential biocontrol activity against a bacterial pathogen of plants. We isolated yeasts from leaves of field-grown pepper plants in South Korea using yeast semi-selective medium containing rifampicin antibiotic to inhibit bacterial growth. Candidate yeasts were selected by greenhouse screening. By screening 838 yeast isolates in a greenhouse, *Pseudozyma churashimaensis* strain RGJ1 was selected as a promising candidate for further field studies. Spray application of strain RGJ1 on pepper seedlings before and after transplanting into the field was very effective for disease suppression of bacterial spot caused by *Xanthomonas axonopodis* pv. vesicatoria. We did not observe direct inhibition of *X. axonopodis* pv. vesicatoria growth on agar medium by strain RGJ1, indicating that RGJ1 may induce systemic resistance in pepper plants to protect against bacterial spot disease. Yeast-mediated induced systemic resistance (ISR) was accompanied by the expression of defense priming genes, including *Capsicum annuum Pathogenesis-Related (CaPR*) 4 for SA/JA signaling and *CaPR5* for ethylene signaling^21^,^37^,^38^,^39^,^40^. Unexpectedly, field spray application of strain RGJ1 on pepper plants significantly reduced the symptoms caused by several viruses, including CMV*, Pepper mottle virus* (PepMoV), *Pepper mild mottle virus* (PMMoV), and *Broad bean wilt virus* (BBWV). To our knowledge, this study is the first report of plant protection against bacterial and viral pathogens mediated by the leaf-colonizing yeast *P. churashimaensis*.

## Results

### Temporal and spatial variation in yeast communities

**Figure 1:**
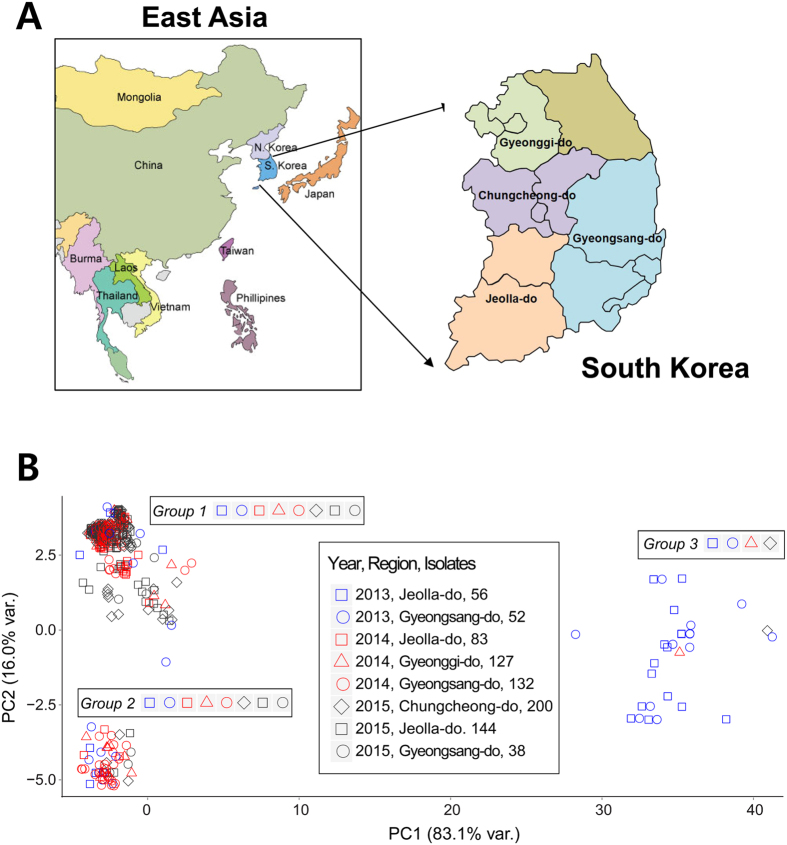
Time and regional distribution of leaf-associated yeasts in South Korea. (**A**) Information about the regional locations where yeasts and yeast-like isolates were collected from pepper leaves in South Korea. The map was obtained from the National Geographic Information Institute in Korea (http://www.ngii.go.kr/child/contents/contentsView.do?rbsIdx=33) and generated by “Adobe Creative Suite 6 Design Standard”. (**B**) Genetic cartography of yeast isolates based on ITS sequence distances. Plot shows the distribution of different yeast strains between samples. Coordinates were calculated and visualized in units of pairwise sequence identity for 832 aligned ITS sequences. Yeast strains per sampling site per year are indicated by different colors and shapes.

Community structure was determined by similarity between internal transcribed spacer (ITS) sequences of 832 yeast isolates, which were sampled during May to October in 2013, 2014, and 2015 because of the growing season of pepper plants under field conditions in Korea (Fig. 1). The distribution of yeast isolates largely sorted into three groups, indicating that the diversity of pepper leaf-colonizing yeasts is limited to a small number of phylotypes. All sampling sites for the years 2013−2015 were covered by the yeast isolates from Group 1 (472 of 832 isolates, 56.7%) and Group 2 (312 of 832, 37.5%). Consequently, the majority of yeasts were clustered together in either Group 1 or 2 regardless of the year and sampling region. In other words, it is highly likely that most yeast strains (more than 94% from both Groups 1 and 2) can be consistently isolated from the sampling sites with little spatiotemporal differences. By contrast, slight temporal and spatial variations were observed by only a few Group 3 yeasts; this group contained the minority of the yeast isolates (only 5.8%), with two isolates in 2013 (Jeolla-do and Gyengsang-Do), one isolate in 2014 (Gyenggi-do), and one isolate in 2015 (Chungcheong-do).

### Strain RGJ1 protects plants against *X. axonopodis* pv. vesicatoria in the greenhouse

**Figure 2:**
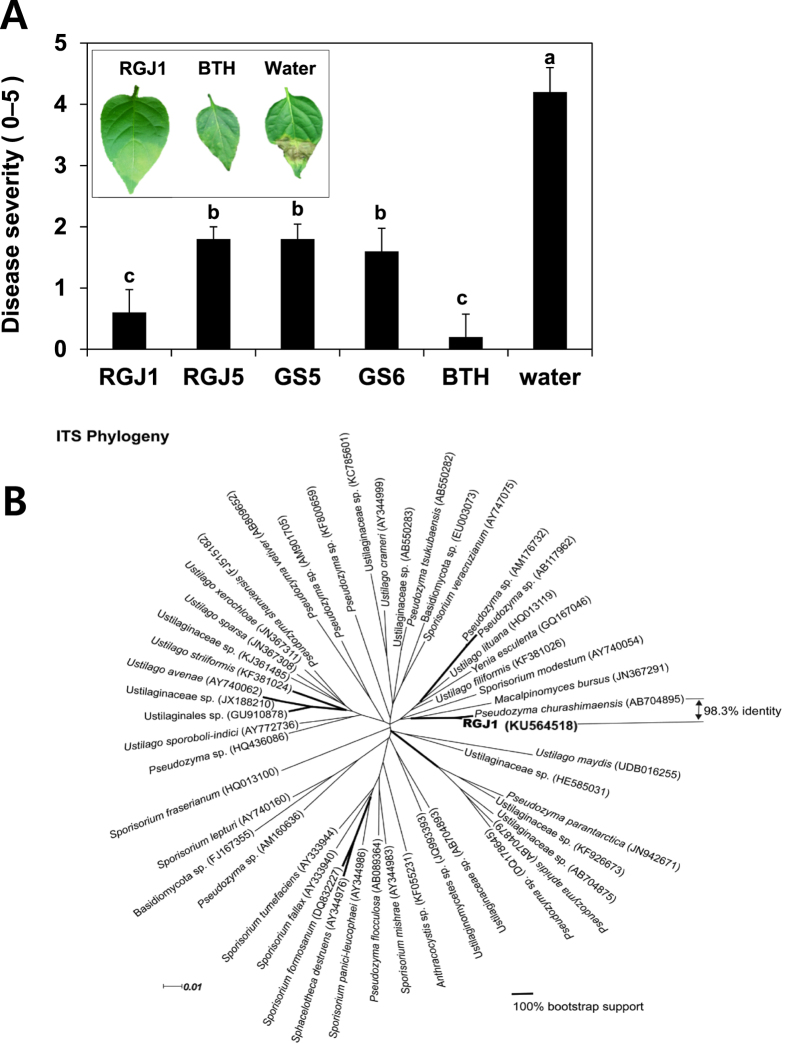
Biological control of *Xanthomonas axonopodis* pv. vesicatoria by *Pseudozyma* spp. in the greenhouse and identification of strain RGJ1. (**A**) The yeast isolates RGJ1, RGJ5, GS5, and GS6 were spray-inoculated on pepper leaves. Pathogen challenge was conducted 1 week after yeast spraying. Disease severity was measured 1 week after leaf infiltration of *X. axonopodis* pv. vesicatoria at OD = 0.001. Bars represent mean ± SEM (*N* = five plants per treatment). The positive control was 0.5 mM BTH. Different letters indicate significant differences between treatments (*P* = 0.05) according to the least significant difference. Experiments were repeated three times with similar results. The inset photograph shows suppression of bacterial spot disease by *X. axonopodis* pv. vesicatoria. The photograph was taken 1 week after pathogen challenge of pepper leaves. (**B**) Unrooted phylogenetic tree of strain RGJ1 and related taxa. Ribosomal ITS sequences were aligned with MUSCLE v3.8.31. Aligned positions with >50% gaps were removed using GBLOCKS v0.91. The phylogeny was inferred with the neighbor-joining method using the Kimura 2-parameter model in PAUP*. Bootstrap support values were determined from 1,000 non-parametric replicates. Branches were highlighted in bold only if they had 100% bootstrap supports. Accession numbers of ITS sequences are indicated between parentheses. The scale represents the number of substitutions per site. The tree was visualized using Dendroscope v3.2.2. The pairwise sequence identity of ITS sequences between RGJ1 and *Pseudozyma churashimaensis* (AB704895) was 98.3%, which represents six mismatches and four gaps in 610 aligned sites.

**Figure 3:**
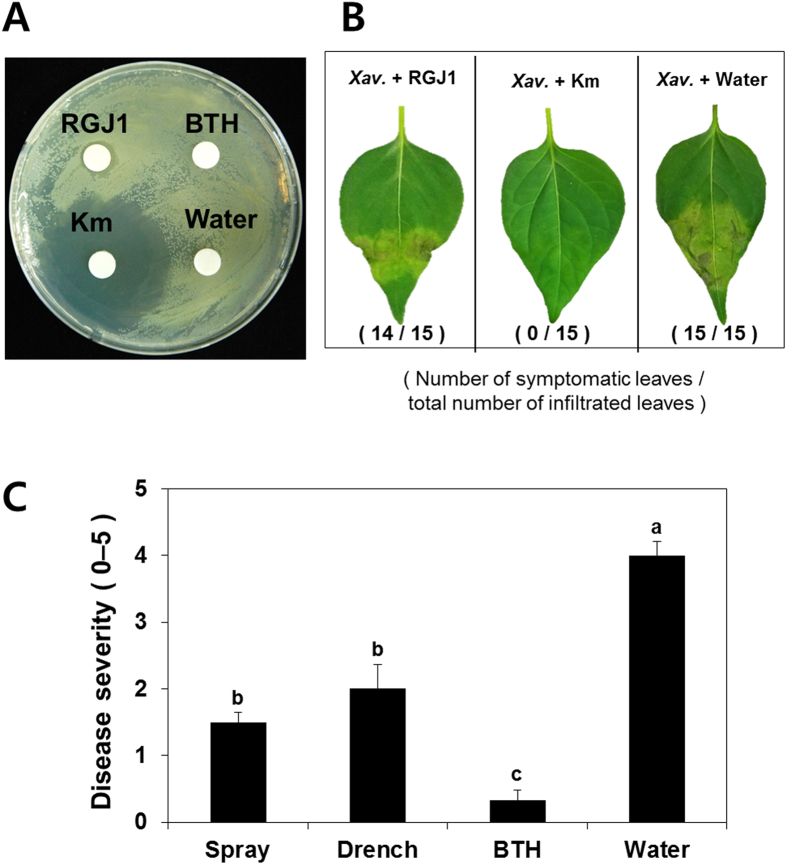
Validation of induced resistance. (**A**) No antagonism displayed between strain RGJ1 and the pathogen *X. axonopodis* pv. vesicatoria. The photo was taken 2 days after inoculation of 20 μL of strain RGJ1 at 10^8^ cfu/mL, 25 μg/mL kanamycin, 20 μL of 1 mM BTH, and water control on a lawn of *X. axonopodis* pv. vesicatoria. (**B**) In-plant assay to validate direct antagonism by strain RGJ1. Xav = *X. axonopodis* pv. vesicatoria; RGJ1 = *Pseudozyma churashimaensis* strain RGJ1; Km = kanamycin The disease symptoms were investigated 7 days after leaf infiltration with three treatments: (1) 1:1 mixture of strain RGJ1 and *X. axonopodis* pv. vesicatoria; (2) 1:1 mixture of strain RGJ1 and 25 μg/mL kanamycin; (3) 1:1 mixture of strain RGJ1 and sterile distilled water. (**C**) Comparison between the different inoculation methods. Strain RGJ1 (10^8^ cfu/mL) was sprayed on leaf or drenched on root. Disease severity was measured 1 week after leaf infiltration of *Xanthomonas axonopodis* pv. vesicatoria at OD = 0.001. Bars represent the mean ± SE (sample size, *N* = 10 replications per treatment). Different letters indicate significant differences between treatments (*P* = 0.05 according to least significant difference).

The primary and secondary screens for yeast-mediated plant protection against *X. axonopodis* pv. vesicatoria infection via foliar spray application resulted in the selection of four yeast and yeast-like isolates. Of these four strains, we selected strain RGJ1 as a model yeast for further analyses. Foliar spray application of strain RGJ1 elicited plant resistance responses at a similar level as treatment with the chemical trigger 0.5 mM benzothiadiazole (BTH) (Fig. 2A). Strain RGJ1 was thought to exert biological control via ISR in plant tissues as RGJ1 did not show an inhibitory effect on *X. axonopodis* pv. vesicatoria growth *in vitro* (data not shown). We tested whether strain RGJ1 conferred ISR in pepper. *X. axonopodis* pv. vesicatoria was tested under greenhouse conditions in Korea. Leaf-spray application of strain RGJ1 reduced disease severity caused by *X. axonopodis* pv. vesicatoria by 60% compared with the untreated control (Fig. 2A). Symptoms of severe necrosis occurred in the control pepper seedlings, but were rarely observed in plants treated with RGJ1 or BTH (Fig. 2A). In pilot experiments, *X. axonopodis* pv. vesicatoria caused symptoms on pepper leaves, and the growth of this pathogen *in vitro* was not affected by co-culture with strain RGJ1 (Fig. 3A). To validate the direct antagonism between yeast and *X. axonopodis* pv. vesicatoria, typical disease symptoms were observed on leaves infiltrated with a 1:1 mixture suspension of two microbes, but were not observed on kanamycin plus *X. axonopodis* pv. vesicatoria treatment (Fig. 3B). Drench application of strain RGJ1 onto roots reduced *X. axonopodis* pv. vesicatoria-mediated disease severity in the leaf by 52%, which is a similar level as that observed after spray application, compared with untreated controls (Fig. 3C). Treatment with 0.5 mM BTH also prevented symptom development in pepper plants infected with *X. axonopodis* pv. vesicatoria (Fig. 3C).

### Identification of strain RGJ1

The unrooted tree based on the neighbor-joining method describes the phylogenetic relationships of strain RGJ1 and 48 closely related taxa (Fig. 2B). The tree topology shows that the ITS sequence of RGJ1 is closest to that of *P. churashimaensis* (GenBank accession AB704895; published in ref. 41), with 100% bootstrap support. Pairwise comparison of ITS sequences only shows 1.7% dissimilarity between RGJ1 and AB704895 (six mismatches and four gaps in 610 aligned positions), indicating that the yeast isolate appears to be taxonomically assigned to *P. churashimaensis*. The ITS sequence of RGJ1 was deposited in GenBank (accession KU564518).

### Defense priming of pathogenesis-related genes

**Figure 4:**
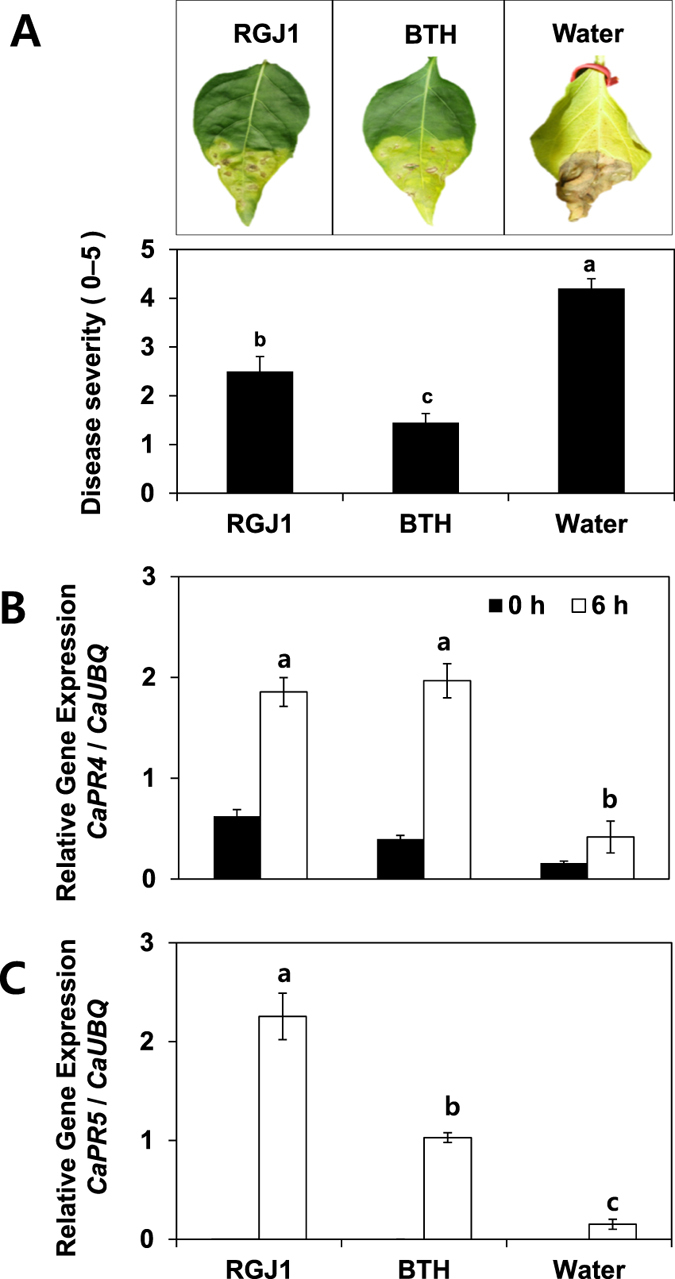
Disease control and defense priming of pepper defense-related genes *CaPR4* and *CaPR5* by strain RGJ1 under field conditions. (**A**) Disease assay 1 week after leaf infiltration application of *Xanthomonas axonopodis* pv. vesicatoria at OD = 0.001 with a needleless syringe. Disease symptoms were evaluated 1 week after infiltration. (**B**) Induction of defense genes by strain RGJ1 on pepper leaves. The expression levels of two defense genes were quantified by qRT-PCR. Transcriptional expression of *CaPR4* and *CaPR5* was evaluated at 0 and 6 h after spray treatment of strain RGJ1, 1 mM BTH, and water on pepper leaves. Bars represent the mean ± SEM (*N* = 4).

Defense priming is an important feature of induced resistance^13^,^21^,^37^,^39^,^42^,^43^. To confirm that foliar spray application of strain RGJ1 elicits plant-induced resistance and defense priming, the expression of the defense-related genes *CaPR4* for SA/JA signaling and *CaPR5* for ethylene signaling after 0 and 6 h of pathogen challenge was examined by quantitative reverse transcription PCR (qRT-PCR) after 0 and 6 h of pathogen challenge under field conditions. The yeast strain RGJ1 caused a 4.5- and 15-fold upregulation in *CaPR4* and *CaPR5* transcription in pepper seedlings 6 h after *X. axonopodis* pv. vesicatoria inoculation. The normalized values of *CaPR4* at 6 h are 1.85 for RGJ1, 1.96 for BTH, and 0.41 for the control (Fig. 4B). The normalized values for *CaPR5* are 2.25 and 0.15 for RGJ1 and the control, respectively (Fig. 4C).

### Plant protection against bacterial and viral pathogens under field conditions

To evaluate whether strain RGJ1 induces ISR under field conditions, we examined plants for symptoms of bacterial spot disease 5–10 days after infection^37^,^39^,^43^. We used a quantitative disease index to determine the disease symptom severity in infected plants that were either mock-treated or treated with RGJ1, or BTH. At 10 dpt, the disease severity in plants treated with strain RGJ1, 1 mM BTH, and water was 2.5, 1.45, and 4.2, respectively (Fig. 4A). Severe leaf disease symptoms appeared in early September and worsened as a consequence of the unusually high temperatures and abundant precipitation in Korea during 2014. Examination of the plants revealed spot, speck, mosaic, and shoe-string patterns, which are characteristic of bacterial spot disease caused by *X. axonopodis* pv. vesicatoria, but also may be caused by CMV infection. In our field study, biological and biochemical assays and PCR analyses identified the causative agent as *X. axonopodis* pv. vesicatoria, which was based on 16S rRNA data, colony color on LB medium, morphology on semi-selective agar media, and a pathogenesis test in pepper plants.

**Figure 5:**
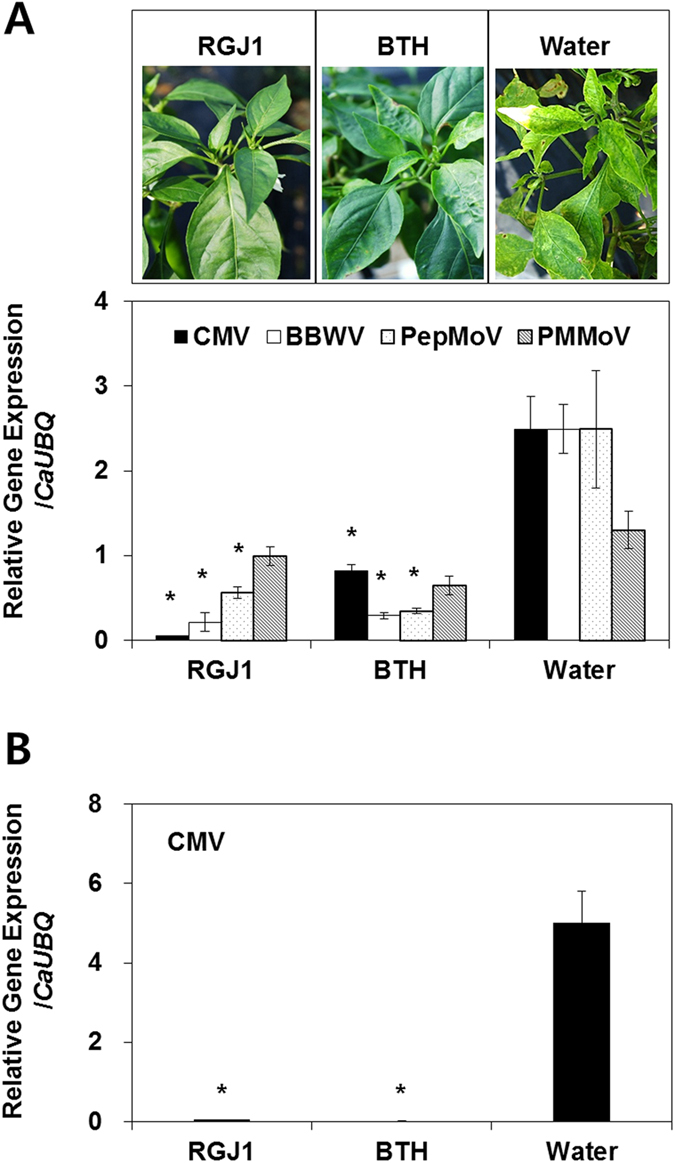
Disease suppression of naturally occurring virus by spray application of strain RGJ1. (**A**) Photo was taken 60 days after transplanting. Disease symptoms caused by naturally occurring mixed virus infection were evaluated. Induced resistance against *Cucumber mosaic virus* (CMV), *Broad bean wilt virus* (BBWV), *Pepper mottle virus* (PepMoV), and *Pepper mild mottle virus* (PMMoV). Disease symptoms caused by naturally occurring CMV were evaluated 134 days post-transplantation (dpt). (**B**) Validation experiment of induced resistance against CMV by foliar application of strain RGJ1 in the greenhouse. The expression of viral-specific genes was measured 60 days after treatment of pepper plants with strain RGJ1, BTH and control. Bars represent the mean value ± SEM (*N* = 5). The housekeeping gene *CaUBQ* was used as a control. The experiment was repeated twice with similar results.

At 60 dpt, the bacterial spot symptoms on pepper plants growing in the field were evaluated again according to the quantitative scale described above^37^,^39^,^43^. Quantification of naturally-occurring CMV, BBWV, PepMoV, and PMMoV by virus-specific primer-based qRT-PCR demonstrated that viral-mediated disease symptoms were significantly reduced by pre- application of strain RGJ1 on pepper leaves. The disease reduction level was similar to that of chemical control via BTH treatment (Fig. 5A, bottom graph). These experiments were validated in the greenhouse (Fig. 5B). The observed CMV accumulation was approximately equivalent to that of the detection limit induced by BTH or strain RGJ1 treatment (Fig. 5B).

### Yield measurements

**Figure 6:**
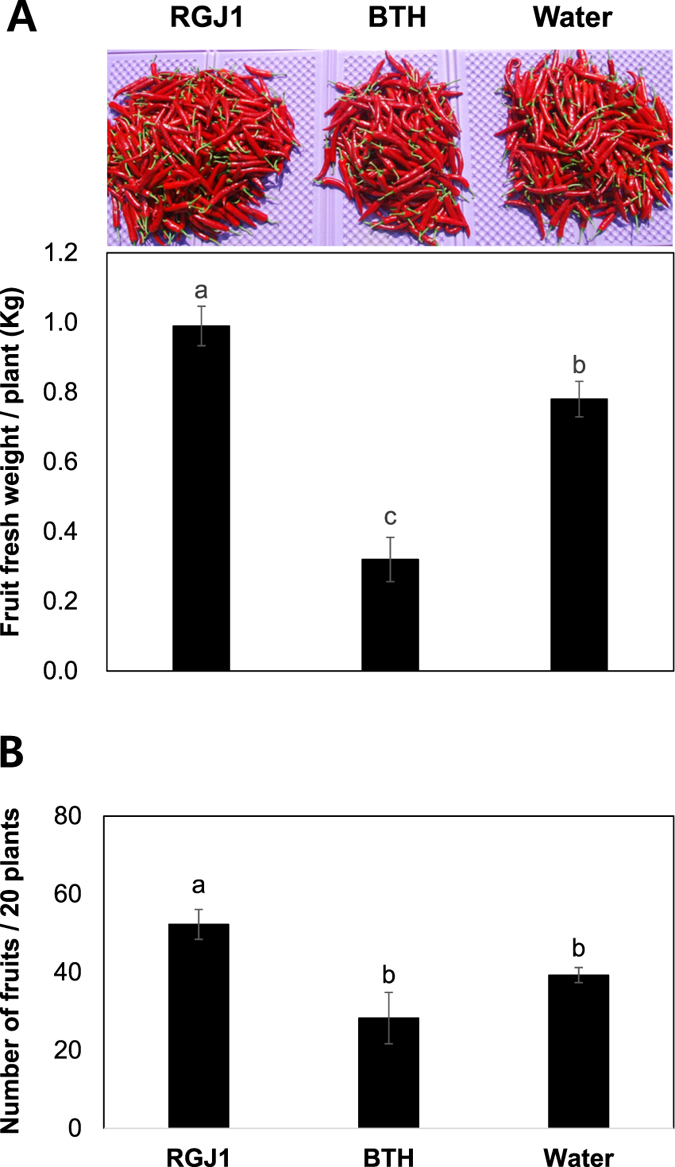
Increase in pepper yield induced by strain RGJ1. (**A**) Fruit fresh weight per plant and (**B**) fruit yield of 20 plants treated with strain RGJ1 or control were assessed 77 days post-transplantation. Water and 1 mM BTH were used as the negative and positive controls, respectively. Different letters indicate statistically significant differences compared with water-treated control plants (*P* = 0.05). Error bars represent mean ± SEM.

At the end of the season, the fruit fresh weight per plant and number of fruits per 20 plants were measured (Fig. 6A,B). Application of strain RGJ1 increased the fruit fresh weight by 1.26-fold compared with the water control (Fig. 6A). However, BTH treatment significantly reduced the fresh fruit weight by 0.58-fold compared with the water control (Fig. 6A). The number of fruits from strain RGJ1 showed a significant 0.34-fold increase compared with that from water control and BTH treatment (Fig. 6B). The fruit yield on plants treated with leaf application of BTH did not differ from that on the water control. The yield increase may be caused by disease suppression by yeast spraying because yeast itself did not show any growth promotion capacity (data not shown).

## Discussion

Many biological and chemical agents have been used to control bacterial and viral pathogens in greenhouses and fields. Extensive studies with beneficial root- and leaf-associated bacteria and fungi show that treatment with beneficial microbes reduces plant symptoms and disease severity after pathogen infection^15^,^16^,^24^,^39^. In this study, we identified a leaf-colonizing yeast that attenuated the development of plant symptoms caused by infection with naturally occurring viruses. The yeast-like fungus *P. churashimaensis* (classified in the *Ustilaginales*) was isolated from leaves of field-grown pepper in South Korea, was successfully re-introduced to pepper leaves, and reduced bacterial and viral disease symptoms that were difficult to control with conventional chemical and cultural control methods. Further mechanistic studies indicated that strain RGJ1 systematically elicited induced resistance in plants. Strain RGJ1 did not directly antagonize the pathogen *in vitro*, but induced expression of the plant systemic resistance marker genes *CaPR4* and *CaPR5*. To our knowledge, this is the first report that foliar spray application of a leaf-colonizing yeast can elicit induced plant resistance against viral pathogens.

Although leaf-inhabiting yeast communities have been studied in rice, sugarcane, moss, and *Ficus* spp. by employing culture-dependent and metagenomics methodologies^44^,^45^,^46^,^47^, yeast diversity on leaves of pepper plants according to the site and year remains unexplored. Here, we conducted a 3-year screen in different locations across South Korea, which yielded 832 leaf-colonizing yeasts and yeast-like isolates. These yeasts and yeast-like isolates were purified using yeast peptone dextrose (YPD) agar medium containing 100 μg/mL rifampicin to inhibit indigenous bacterial growth. While many colonies with very diverse morphologies and colors were observed on the medium, comparison of the fungal ITS sequences along the principal component axes showed that the 832 yeast isolates are classified into only three groups (Fig. 1). A few isolates (5.8%) representing Group 3 appeared specifically in Jeolla-do (2013), Gyeongsang-do (2013), Gyeonggi-do (2014), and Chungcheong-do (2015), indicating that the minority of the yeasts can be influenced by spatiotemporal changes. However, the majority of the yeast isolates (over 94%) belong to either Group 1 or 2, each of which is represented in all eight sampling conditions according to the site and year (Fig. 1). This pattern shows that most yeast isolates of pepper leaves are not greatly associated with spatiotemporal changes and that the two groups appear consistently, regardless of the sampling site and year. In fact, the limited effect of spatial and temporal changes was also documented in a previous study of epiphytic yeast diversity in rice phyllospheres using the restriction fragment length polymorphism pattern and sequence of the D1/D2 region of a ribosomal RNA gene^46^. However, the yeast species colonizing foliar surfaces vary according to the host plants^48^. Therefore, how much spatiotemporal changes affect yeast communities in other host plants remains unresolved and further analyses are required. The abundance of *Pseudozyma* spp. on rice and sugarcane in Thailand was detected by extracting DNA from leaf washing samples and amplifying the D1/D2 domain of the large subunit rRNA gene sequence^46^,^47^. The yeasts and yeast-like isolates were tested in greenhouse screens for their ability to confer pepper plant protection against *X. axonopodis* pv. vesicatoria. We selected strain RGJ1 because it strongly and consistently reduced disease symptoms from viral infections during repeated experiments.

Our objective during the initial stage of this study was to identify a new biological agent to trigger plant ISR against notorious foliar pathogens. The first criterion to meet the objective was a leaf-colonizing behavior. The selected strain RGJ1 is an aggressive leaf colonizer that maintains more than 10^5^ cells/leaf disc (diameter = 1 cm) up to 30 days after spray application (data not shown). Yeasts have been used as biological control agents for postharvest diseases; they are a promising alternative to chemical fungicides and meet stringent regulations for crop food safety^49^. The mechanism of postharvest biological control was proposed to include induction of host defense, pathogen antagonism due to lytic enzyme secretion, alleviation of oxidative damage, and biofilm formation^49^. Only recently, some studies reported that foliar spray of epiphytic yeast protects tomato, cucumber, and potato plants against fungal and bacterial pathogens^34^,^35^,^36^,^50^.

ISR in plants is an attractive means to control foliar pathogens due to long-term effectiveness against a broad spectrum of pathogens^14^,^16^,^51^. In our system, it is difficult to exclude the spatial separation between inoculant and pathogen, which is an important criterion for ISR, because a liquid suspension of yeast was sprayed on the leaf and the bacteria pathogen *X. axonopodis* pv. vesicatoria was infiltrated into the same leaf. A direct inhibition of *X. axonopodis* pv. vesicatoria by strain RGJ1 in pepper leaves was not detected *in vitro* on agar medium or during a leaf-infiltration assay with a 1:1 mixture of the two microbes, indicating that yeast-mediated plant protection is caused by elicitation of ISR (Fig. 3). Leaf application of *Pseudozyma aphidis* induced systemic resistance against the bacterial canker pathogen *Clavibacter michiganensis* on tomato, the powdery mildew fungus *Podosphaera xanthii* on cucumber, and the gray mold pathogen *Botrytis cinerea* on *Arabidopsis thaliana*^35^,^36^. However, the authors demonstrated direct inhibition of target pathogen growth by *P. aphidis* crude extract. These results suggest that *P. aphidis* may mediate both ISR and antagonism as mechanisms of biological control. Our results clearly showed that the yeast isolates only elicited plant systemic defense mechanisms rather than metabolite-mediated antagonism or nutrient competition (Figs 2A and 3). The defense priming of *CaPR4* and *CaPR5* expression by strain RGJ1 supports the elicitation of ISR (Fig. 4B and C). *P. aphidis* triggers ISR independently of SA, JA, and *non-expressor of PR1 (NPR1*) in *Arabidopsis*, and via SA-independent signaling in tomato, as determined using plant mutants impaired in SA and JA signaling or transgenic plants expressing the SA hydroxylase *NahG*. To determine the detailed ISR signaling network, knock-down systems such as virus-induced gene silencing (VIGS) can be utilized in pepper because the knock-out mutants of defense signaling are not available.

While conducting the field trials, we observed significantly fewer viral symptoms such as mosaic leaf, shoe-string patterns or leaf shape deformation on yeast-treated plants than on water-treated controls (Fig. 5A). The observation led us to quantify virus contents. In South Korea, pepper plants in the field generally exhibit mixed infections by various viruses including CMV and PMMoV^39^. The four viruses tested in the current study accumulated to similar levels in plants treated with the chemical SAR trigger BTH as in plants treated with strain RGJ1 (Fig. 5A, below graph). This result was validated in a greenhouse experiment with CMV (Fig. 5B). As we mentioned above, this is the first demonstration of yeast-elicited ISR conferring plant protection from a viral pathogen. Previous work showed that root application of bacteria and fungi in the greenhouse and field elicited ISR against viruses^14^,^16^. The new and re-emerging viral diseases appear to be potentiated by climate change around the world including East Asia^6^,^52^. However, efficient control agents against plant virus are not available, except for a few virus-resistant plants produced by breeding programs. Biologically induced ISR is an attractive mechanism to manage plant viral infections. We showed that foliar application of a leaf-colonizing yeast can provide a means to control plant viral disease under field conditions.

In conclusion, our study provides new information on epiphytic yeast-mediated plant ISR against viral pathogens under field conditions. Future research will identify yeast determinant(s) that elicit ISR. Previous studies with *Pseudozyma* spp. primarily isolated plant growth-promoting factors such as indole-3-acetic acid^53^,^54^. However, many studies reported that IAA modulated plant defense and enhanced plant susceptibility to disease^55^,^56^. Possible alternative candidates could be extracellular biosurfactants, which are lipids and byproducts secreted by *Pseudozyma* spp.^57^,^58^,^59^. Lipid-mediated plant systemic defense has been characterized in many plant species^60^,^61^. Cell wall components also could be targets of the yeast determinant(s) to elicit induced resistance^62^,^63^. In our preliminary experiment, culture filtrate was not sufficient to elicit ISR, indicating that yeast cells were required (data not shown). These combined results suggest that yeast-elicited induced resistance can be utilized in disease management programs to protect against viruses and promote crop yield.

## Methods

### Yeast isolation from leaves of field-grown pepper plants in South Korea

Yeasts were isolated from leaves of field-grown pepper plants cultivated in South Korea during May to October in 2013, 2014, and 2015. Two to three leaves from a healthy plant were detached and placed in a plastic bag in an icebox for preservation during transport to the laboratory. Yeasts were isolated from leaf surfaces by sonication and from inside leaves by homogenizing leaves after surface sterilization in 0.1 M MgSO_4_ and preparing serial dilutions. Then, the serial dilutions were plated on YPD agar medium (10 g of yeast extract, 20 g of peptone, 20 g of dextrose, and 20 g of agar per 1 L of water) containing 100 μL/mL rifampicin to inhibit prokaryotic bacterial growth. Two days after plating, individual isolates were transferred to fresh YPD agar medium to confirm their purity in order to avoid cross contamination by streaking. A total of 838 yeasts and yeast-like isolates were purified and selected for further study.

### Metagenomic analysis of leaf yeast microbiota

Total genomic DNA was extracted from the purified isolates using an AccuPrep^®^ Genomic DNA Extraction Kit (Bioneer, Daejeon, Korea). The nuclear ribosomal ITS region of genomic DNA was amplified with ITS1 (5′-TCCGTAGGTGAACCTGCGG-3′) and ITS4 (5′-TCCTCCGCTTATTGATATGC-3′) primers using Quick PCR Premix containing Taq DNA polymerase, dNTPs, reaction buffer, and tracking dye (Genenmed, Daejeon, Korea). PCR analyses were conducted in a PTC100 Thermal Cycler (MJ Research, Watertown, MA, USA) using an initial denaturation step of 95 °C for 5 min; followed by 29 cycles of denaturation for 1 min at 94 °C, primer annealing for 30 s at 52 °C, and extension for 30 s at 72 °C; with a final extension for 10 min at 72 °C. Amplified PCR products were detected by electrophoresis on a 0.75% agarose gel and purified with an AccuPrep^®^ PCR Purification Kit (Bioneer). The ITS region of the yeast isolates was sequenced using the same PCR primers and the ABI3700 automated DNA sequencer (Applied Biosystems, Foster City, CA, USA). The ITS sequences of yeast isolates were aligned using MUSCLE v3.8.31 (Edgar, 2004) with default penalties for gaps. Aligned positions with >50% gaps were removed using GBLOCKS v0.91^64^. BioEdit v7.1.3^65^ was used to exclude ambiguous and uninformative variable sites at both ends of the alignment. Then, pairwise sequence distances from the resulting alignment were calculated using PAUP* v4.0b10^66^ with the Kimura 2-parameter model, and a distance matrix was prepared. To distribute the yeast isolates according to the ITS sequence similarity, principal component analysis and dot plotting were performed using the functions PRCOMP and GGPLOT2, respectively, of the R package v3.2.2. The phylogenetic position of the strain RGJ1 was then determined. The DNA sequence was BLAST-searched against the ITS sequence database UNITE^67^. The ITS sequences of RGJ1 and related taxa were aligned and edited using the methods described above. Phylogenetic analysis was performed based on the neighbor-joining method^68^. Tree reconstruction was conducted with PAUP* v4.0b10 with the Kimura 2-parameter model. Phylogenetic confidence was evaluated by the non-parametric bootstrap method with 1,000 replicates^69^. Bootstrap tree consensus was obtained using the Python library SumTrees with the option of 50% majority rule^70^. Trees were visualized using Dendroscope v2.7.4^71^.

### Induced resistance against *X. axonopodis* by yeasts in the greenhouse

A total of 838 yeasts and yeast-like isolates were purified during a 3-year screen of plant protection against *X. axonopodis* pv. vesicatoria, including 108 isolates from 2013, 342 isolates from 2014, and 382 isolates from 2015, using a modified protocol that was described previously^43^. Four isolates were ultimately selected because they consistently protected plants from *X. axonopodis* pv. vesicatoria, including *P. churashimaensis* RGJ1, *Cryptococcus magnus* RGJ5, *Pseudozyma aphidis* GS5, and *Pseudozyma tsukubaensis* GS6. Purified yeast isolates were grown on YPD agar medium for 2–3 days at 30 °C, yeast suspensions were prepared by harvesting isolated yeast colonies into sterile distilled water by centrifugation at 10,000 × *g* and washed once with sterile distilled water, and the yeast suspension density was adjusted to 10^8^ colony-forming units (cfu)/mL.

Pepper plants were grown and disease assays were performed as described previously^39^,^72^. Plants were grown in a controlled environment growth room at 25 ± 2 °C under fluorescent light with an intensity of approximately 7,000 lux and a 12 h/12 h day/night cycle. Briefly, pepper (*C. annuum*) seeds were surface-sterilized with 6% sodium hypochlorite, washed four times with sterile distilled water, and then maintained on Murashige and Skoog agar medium (Duchefa, Haarlem, The Netherlands) at 25 °C for 3 days until germination. Germinated seeds were then transplanted into soil-less medium (Punong Horticulture Nursery Media LOW, Punong Co. LTD, Gyeongju, Korea). Seedlings were grown at 25 ± 2 °C under fluorescent light in the controlled environment growth room (12 h/12 h day/night cycle, 7,000 lux light intensity), and then transferred to the KRIBB greenhouse facility in Daejeon, South Korea. The experiment was repeated three times with 12 replications (one plant per replication and three leaves per plant).

Then, the foliar part of 4-week-old pepper seedlings was sprayed until drop-off with 20 mL of a suspension (10^8^–10^9^ cfu/mL) of each yeast strain. The positive control was 0.5 mM BTH (Syngenta, Durham, NC, USA), which elicits SAR to bacterial and viral pathogens^73^,^74^,^75^. The negative control was sterile distilled water. The leaves were infiltrated with *X. axonopodis* pv. vesicatoria (OD_600_ = 0.01) 1 week after spraying yeast. The severity of symptoms on the leaf was scored from 0 to 5 as described previously^39^: 0 = no symptoms, 1 = mild chlorosis, 2 = chlorosis only, 3 = chlorosis and mild necrosis, 4 = necrosis, and 5 = severe necrosis of the inoculated area. Three leaves were infiltrated with *X. axonopodis* pv. Vesicatoria and disease severity was evaluated 5–7 days later. For long-term maintenance, yeast strains were preserved in YPD broth containing 15% glycerol (v/v) and stored at −70 °C.

### Confirmation of induced resistance by excluding direct antagonism of yeast *in vitro* and *in planta*

To test whether yeast strain RGJ1 directly inhibits *X. axonopodis* pv. vesicatoria growth, bioassays were performed using the paper disc assay method^76^. One hundred microliters of 10^8^ cfu/mL *X. axonopodis* pv. vesicatoria suspension was spread onto LB agar medium. Fifty microliters of 10^8^ cfu/mL strain RGJ1 suspension was pipetted onto paper discs. The paper discs were transferred aseptically onto the surface of the growth medium. Two days later, the development of the zones of inhibition was checked. Kanamycin (25 μL/mL) was used as a positive control. A minimum of three replicate plates were prepared for the assay. To test the direct inhibitory effects of strain RGJ1 *in planta*, a 1:1 mixture suspension of *X. axonopodis* pv. vesicatoria and *P. churashimaensis* strain RGJ1 at the same concentrations (OD_600_ = 0.05) was infiltrated into pepper leaves as described above. A 1:1 mixture suspension of *X. axonopodis* pv. vesicatoria and 25 μL/mL kanamycin was used as a positive control. Next, to confirm ISR, different inoculation methods were employed including drench application, which indicates spatial separation between yeast on roots at OD_600_ = 1 and the pathogen on leaves, and disease severity was assessed after 5–7 days.

### Field trial

The field trial was conducted at Geumsan-gun, Chungcheongnam-do, Korea (36°35′ 32.27″North, 127°30′ 34.75″East) from the first week of April to the third week of September in 2014. To test ISR under field conditions, pepper seedlings were sprayed with each yeast isolate suspension at 10^8^–10^9^ cfu/mL and/or 1 mM BTH solution, and transplanted at a distance of 40 cm apart in the field. Sterilized water was used as a negative control. Before transplanting, each field row was covered with black and white polyethylene plastic film. Treated pepper plants were grown in beds with a height of 20 cm and an area of 50 × 800 cm. Single-row treatment plots containing 20 plants were replicated four times in a completely randomized design. For disease assessment, disease severity (0–5) was evaluated at 10 and 60 dpt as described above. To assess qRT-PCR analysis, four replications per treatment were used. One replication included 12 leaves (three leaves per plant × four plants) from one block. To assess leaf colonization of strain RGJ1, the leaf sprayed with strain RGJ1 was collected and the yeast population density of the leaf was measured by the dilution-plating method.

### Assessment of defense priming of PR genes by qRT-PCR

qRT-PCR was performed using a Bio-Rad CFX96 instrument. Total RNA was isolated from pepper leaf tissues using Tri reagent (Molecular Research Inc., Cincinnati, OH, USA) according to the manufacturer’s instructions and as described in our previous study^37^. First-strand cDNA synthesis was performed with 2 μg of DNase-treated total RNA, oligo-dT primers, and Moloney murine leukemia virus reverse transcriptase (Enzynomics, Daejeon, Korea). qRT-PCR assays consisted of cDNA, iQ™ SYBR^®^ Green Supermix (Bio-Rad Inc., Hercules, CA, USA), and 10 pM of each primer. Cycling parameters were as follows: initial polymerase activation for 10 min at 95 °C; and then 40 cycles of 30 s at 95 °C, 60 s at 55 °C, and 30 s at 72 °C. Conditions were determined by comparing threshold values in a dilution series of the product with those of a non-reverse transcriptase template control and a non-template control for each primer pair. The expression of candidate priming genes was analyzed using the following primer pairs: 5′-AACTGGGATTTGAGAACTGCCAGC-3′ (*CaPR4*-F) and 5′-ATCCAAGGTACATATAGAGCTTCC-3′ (*CaPR4*-R); 5′-CTCCACAAGAAACAAGGCA-3′ (*CaPR5*-F) and 5′-GTACGAAGCACGCACACAA-3′ (*CaPR5*-R). *CaUBQ* (ubiquitin) was analyzed using the primers 5′-GCACAAGCACAAGAAGGTTAAG-3′ (forward) and 5′-GCACCACACTCAGCATTAGGA-3′ (reverse) as a loading control to ensure that equal RNA amounts were used in each assay. Relative transcript quantification was calculated using the 2-ΔΔCT method. Standard error of mean values among replicates were calculated using Bio-Rad manager (version 2.1) (Bio-Rad CFX Connect). The Student’s *t*-test was computed to determine statistically significant differences between treated and untreated samples. If *P*-values were <0.05, the target genes were considered to be differentially expressed. Relative transcript abundance was normalized with respect to *CaUBQ* mRNA levels.

### Diagnosis of viral diseases by qRT-PCR in the field trials

For viral diagnosis, test samples were selected from areas of the plant that exhibited disease symptoms. Samples were ground in 50 mM NaHPO_4_ (pH 7.0) buffer. To confirm virus infection, an RT-PCR technique was employed using specific primers for CMV coat protein [5′-CGTTGCCGCTATCTCTGCTAT-3′ (forward) and 5′-GGATGCTGCATACTGACAAACC-3′ (reverse)], BBWV [5′-AATGAAGTGGTGCTCAACTACACA-3′ (forward) and 5′-TTTTGGAGCATTCAACCATTTGGA-3′ (reverse)], PepMoV [5′-AAGATCAGACACATGGA-3′ (forward) and 5′-CAAGCAAGGGTATGCATGT-3′ (reverse)], and PMMoV [5′-ACAGTTTCCAGTGCCAATCA-3′ (forward) and 5′-AAGCGTCTCGGCAGTTG-3′ (reverse)].

### Validation experiment for ISR against CMV in the greenhouse

To validate ISR against viruses under field conditions, the effect of yeast strain RGJ1 on ISR against CMV in the greenhouse following artificial challenge of CMV was evaluated. Four-week-old pepper plants were used to validate CMV infection under greenhouse conditions. Three of the first four leaves were rub-inoculated with CMV 7 days after spray inoculation of strain RGJ1 at OD_600_ = 1 as described previously^39^,^77^. The Fny strain of CMV was obtained from Plant Virus GenBank in Korea (http://knrrb.knrrc.or.kr/index.jsp?rrb=pvgb). Virus quantification was performed by qRT-PCR as described above at 2 weeks after virus inoculation.

### Fruit yield measurement

To measure the fruit yield, the fruit fresh weight per plant and number of pepper fruits per 20 plants in a row were measured at 16 weeks after transplanting with four replications. Only red-colored fruits were harvested for market value.

### Statistical analysis

An analysis of variance for experimental data sets was performed using JMP software version 5.0 (SAS Institute Inc., Cary, NC, USA). Significant effects of treatments were determined by the magnitude of the *F*-value (*P* = 0.05). When a significant *F*-value was observed, separation of means was accomplished by Fisher’s protected least significant difference at *P* = 0.05.

## Additional Information

**How to cite this article**: Lee, G. *et al*. Foliar application of the leaf-colonizing yeast *Pseudozyma churashimaensis* elicits systemic defense of pepper against bacterial and viral pathogens. *Sci. Rep.* **7**, 39432; doi: 10.1038/srep39432 (2017).

**Publisher's note:** Springer Nature remains neutral with regard to jurisdictional claims in published maps and institutional affiliations.

## References

[b1] Agrios G. N. Plant pathology in the 20^th^ century In Plant Pathology (ed. Agrios S. G.) 45–75 (Academic Press, 2005).

[b2] Lamberth C., Jeanmart S., Luksch T. & Plant A. Current challenges and trends in the discovery of agrochemicals. Science, 341, 742–746 (2013). 23950530 10.1126/science.1237227

[b3] Enserink M. . Smarter pest control: The pesticide paradox. Science 341, 728–729 (2013). 23950523 10.1126/science.341.6147.728

[b4] Köhler H. R. & Triebskorn R. Wildlife ecotoxicology of pesticides: can we track effects to the population level and beyond? Science 341, 759–765 (2013). 23950533 10.1126/science.1237591

[b5] Malakof D. & Stokstad E. Pesticide planet. Science 341, 730–731 (2013). 23950524 10.1126/science.341.6147.730

[b6] Scholthof K. B. G. . Top 10 plant viruses in molecular plant pathology. Mol. Plant. Pathol. 12, 938–954 (2011). 22017770 10.1111/j.1364-3703.2011.00752.x PMC6640423

[b7] Kupferschmidt K. A lethal dose of RNA. Science 341, 732–733 (2013). 23950525 10.1126/science.341.6147.732

[b8] Murphy J. F. Applied aspects of induced resistance to plant virus infection In Natural Resistance Mechanisms of Plants to Viruses (ed. Loebenstein G. & Carr J. P.) 1–11 (Springer, 2006).

[b9] Fu Z. Q. & Dong X. Systemic acquired resistance: Turning local infection into global defense. Annu. Rev. Plant Biol. 64, 839–863 (2013). 23373699 10.1146/annurev-arplant-042811-105606

[b10] Borges A. A. & Sandalio L. M. Induced resistance for plant defense. Front. Plant Sci. 6, 109 (2015). 25759706 10.3389/fpls.2015.00109 PMC4338655

[b11] Ross A. F. Systemic acquired resistance induced by localized virus infections in plants. Virology 14, 340–358 (1961). 13743578 10.1016/0042-6822(61)90319-1

[b12] Tally A. . Commercial development of elicitors of induced resistance to pathogens. In Induced plant defenses against pathogens and herbivores: Biochemistry, Ecology, and Agriculture (ed. Agrawal A. A., Tuzun S. & Bent E.) 357–369 (APS press, 1999).

[b13] van Hulten M. . Costs and benefits of priming for defense in *Arabidopsis*. Proc. Natl. Acad. Sci. USA 103, 5602–5607 (2006). 16565218 10.1073/pnas.0510213103 PMC1459400

[b14] Kloepper J. W., Ryu C. M. & Zhang S. Induced systemic resistance and promotion of plant growth by *Bacillus* spp. Phytopathology 94, 1259–1266 (2004). 18944464 10.1094/PHYTO.2004.94.11.1259

[b15] Faoro F. & Gozzo F. Is modulating virus virulence by induced systemic resistance realistic? Plant Sci. 234, 1–13 (2015). 25804804 10.1016/j.plantsci.2015.01.011

[b16] Pieterse C. M. J. . Induced systemic resistance by beneficial microbes. Annu. Rev. Phytopathol. 52, 347–375 (2014). 24906124 10.1146/annurev-phyto-082712-102340

[b17] Jetiyanon K. & Kloepper J. W. Mixtures of plant growth-promoting rhizobacteria for induction of systemic resistance against multiple plant diseases. Biol. Control 24, 285–291 (2002).

[b18] Wei G., Kloepper J. W. & Tuzun S. Induced systemic resistance to cucumber diseases and increased plant growth by plant growth-promoting rhizobacteria under field conditions. Phytopathology 86, 221–224 (1996).

[b19] Zehnder G. W., Murphy J. F., Sikora E. J. & Kloepper J. W. Application of rhizobacteria for induced resistance. Eur. J. Plant Pathol. 107, 39–50 (2001).

[b20] Murphy J. F. . Rhizobacteria-mediated growth promotion of tomato leads to protection against *Cucumber mosaic virus*. Phytopathology 93, 1301–1307 (2003). 18944330 10.1094/PHYTO.2003.93.10.1301

[b21] Yi H. S. . Benzothiadiazole-elicited defense priming and systemic acquired resistance against bacterial and viral pathogens of pepper under field conditions. Plant Biotechnol. Rep. 6, 373–380 (2012).

[b22] Zehnder G. W. . Induction of resistance in tomato against cucumber mosaic cucumovirus by plant growth-promoting rhizobacteria. Bio. Control 45, 127–137 (2000).

[b23] Murphy J. F. . Plant growth-promoting rhizobacterial mediated protection in tomato against *Tomato mottle virus*. Plant Dis. 84, 779–784 (2000). 10.1094/PDIS.2000.84.7.779 30832108

[b24] Jetiyanon K., Fowler W. D. & Kloepper J. W. Broad-spectrum protection against several pathogens by PGPR mixtures under field conditions in Thailand. Plant Dis. 87, 1390–1394 (2003). 10.1094/PDIS.2003.87.11.1390 30812559

[b25] Paulitz T. C. & Belanger R. R. Biological control in greenhouse systems. Annu. Rev. Phytopathol. 39, 103–133 (2001). 11701861 10.1146/annurev.phyto.39.1.103

[b26] Avis T. J. & Bélanger R. R. Mechanisms and means of detection of biocontrol activity of *Pseudozyma* yeasts against plant-pathogenic fungi. FEMS Yeast Res. 2, 5–8 (2002). 12702315 10.1111/j.1567-1364.2002.tb00062.x

[b27] Jarvis W. R., Shaw L. A. & Traquair J. A. Factors affecting antagonism of cucumber powdery mildew by *Stephanoascus flocculosus* and *S. rugulosus*. Mycol. Res. 92, 162–165 (1989).

[b28] Hajlaoui M. R. & Bélanger R. R. Comparative effects of temperature and humidity on the activity of three potential antagonists of rose powdery mildew. Eur. J. Plant Pathol. 97, 203–208 (1991).

[b29] Hajlaoui M. R. & Bélanger R. R. Antagonism of the yeast-like phyllophane fungus *Sporothrix flocculosa* against *Erysiphe graminis* var. tritici. Biocontrol Sci. Technol. 3, 427–434 (1993).

[b30] Kiss L. A review of fungal antagonists of powdery mildews and their potential as biocontrol agents. Pest Manag. Sci. 59, 475–483 (2003). 12701710 10.1002/ps.689

[b31] Hajlaoui M. R., Benhamou N. & Bélanger R. R. Cytochemical study of the antagonistic activity of *Sporothrix flocculosa* on rose powdery mildew *Sphaerotheca pannosa* var. *rosae*. Cytol. Histol. 82, 583–589 (1992).

[b32] Hajlaoui M. R., Traquair J. A., Jarvis W. R. & Bélanger R. R. Antifungal activity of extracellular metabolites produced by *Sporothrix flocculosa*. Biocontrol Sci. Technol. 4, 229–237 (1994).

[b33] Hammami W. . Ecological basis of the interaction between *Pseudozyma flocculosa* and powdery mildew fungi. Appl. Environ. Micobiol. 77, 926–933 (2011). 10.1128/AEM.01255-10 PMC3028749 21115715

[b34] Buxdorf K., Rahat I. & Levy M. *Pseudozyma aphidis* induces ethylene-independent resistance in plant. Plant Signal. Behav. 8, e26273 (2013). 23989134 10.4161/psb.26273 PMC4106499

[b35] Barda O. . *Pseudozyma aphidis* induces salicylic-acid-independent resistance to *Clavibacter michiganensis* in tomato plants. Plant Dis. 99, 621–626 (2015). 10.1094/PDIS-04-14-0377-RE 30699688

[b36] Gafni A. . Biological control of the cucurbit powdery mildew pathogen *Podosphaera xanthii* by means of the epiphytic fungus *Pseudozyma aphidis* and parasitism as a mode of action. Front Plant Sci. 6, 132 (2015). 25814995 10.3389/fpls.2015.00132 PMC4356082

[b37] Lee B., Lee S. & Ryu C. M. Foliar aphid feeding recruits rhizosphere bacteria and primes plant immunity against pathogenic and non-pathogenic bacteria in pepper. Ann. Bot. 110, 281–290 (2012). 22437662 10.1093/aob/mcs055 PMC3394643

[b38] Shin R., Kim M. J. & Paek K. H. The *CaTin1(Capsicum annuum TMV-induced* Clone 1) and *CaTin1–2* genes are linked head-to-head and share a bidirectional promoter. Plant Cell Physiol. 44, 549–554 (2003). 12773642 10.1093/pcp/pcg069

[b39] Song G. C., Choi H. K. & Ryu C. M. The folate precursor para-aminobenzoic acid elicits induced resistance against *Cucumber mosaic virus* and *Xanthomonas axonopodis*. Ann. Bot. 111, 925–934 (2013). 23471007 10.1093/aob/mct049 PMC3631333

[b40] Yang J. W. . Whitefly infestation of pepper plants elicits defence responses against bacterial pathogens in leaves and roots and changes the below-ground microflora. J. Ecol. 99, 46–56 (2011).

[b41] Morita T. . Isolation and screening of glycolipid biosurfactant producers from sugarcane. Biosci. Biotechnol. Biochem. 76, 1788–1791 (2012). 22972331 10.1271/bbb.120251

[b42] van Wees S. C., Van der Ent S. & Pieterse C. M. J. Plant immune responses triggered by beneficial microbes. Curr. Opin. Plant Biol. 11, 443–438 (2008). 18585955 10.1016/j.pbi.2008.05.005

[b43] Yang J. W., Yu S. H. & Ryu C.-M. Priming of defense-related genes confers root-colonizing bacilli-elicited induced systemic resistance in pepper. Plant Pathology J. 25, 303–440 (2009).

[b44] Voriskova J. & Baldrian P. Fungal community on decomposing leaf letter undergoes rapid successional changes. ISME J. 7, 477–486 (2013). 23051693 10.1038/ismej.2012.116 PMC3578564

[b45] Kachalkin A. V. & Yurkov A. M. Yeast communities in *Sphagnum* phyllosphere along the temperature-moisture ecocline in the boreal forest-swamp ecosystem and description of *Candida sphagnicola* sp. nov. Antonie Van Leeuwenhoek 102, 29–43 (2012). 22331450 10.1007/s10482-012-9710-6

[b46] Nasanit R., Krataithong K., Tantirungkij M. & Limtong S. Assessment of epiphytic yeast diversity in rice (*Oryza sativa*) phyllosphere in Thailand by a culture-independent approach. Antonie Van Leeuwenhoek 107, 1475–1490 (2015a). 25842038 10.1007/s10482-015-0442-2

[b47] Nasanit R., Tangwong-O-Thai A., Tantirungkij M. & Limtong S. The assessment of epiphytic yeast diversity in sugarcane phyllosphere in Thailand by culture-independent method. Fungal Biol. 119, 1145–1157 (2015b). 26615738 10.1016/j.funbio.2015.08.021

[b48] Arnold A. E. & Lutzoni F. Diversity and host range of foliar fungal endophytes: Are tropical leaves biodiversity hostpots? Ecology 88, 541–549 (2007). 17503580 10.1890/05-1459

[b49] Liu J. . Review: Utilization of antagonistic yeasts to manage postharvest fungal diseases of fruit. Int. J. Food Microbiol. 167, 153–160 (2013). 24135671 10.1016/j.ijfoodmicro.2013.09.004

[b50] Hadwiger L. A., McDonel H. & Glawe D. Wild yeast strains as prospective candidates to induce resistance against potato late blight (*Phytophthora infestans*). Am. J. Potato Res. 92, 378–386 (2015).

[b51] van Wees S. C. . Enhancement of induced disease resistance by simultaneous activation of salicylate- and jasmonate-dependent defense pathways in *Arabidopsis thaliana*. Proc Natl. Acad. Sci. USA 97, 8711–8716 (2000). 10890883 10.1073/pnas.130425197 PMC27013

[b52] Jones R. A. C. Plant virus ecology and epidemiology: historical perspectives, recent progress and future prospects. Ann. Appl. Biol. 164, 320–347 (2014).

[b53] Fu S. F. . Plant growth-promoting traits of yeasts isolated from the phyllosphere and rhizosphere of *Drosera spatulata* Lab. Fungal Biol. 120, 433–448 (2016). 26895872 10.1016/j.funbio.2015.12.006

[b54] Sun P. F. . Indole-3-acetic acid-producing yeasts in the phyllosphere of the Carnivorous plant *Drosera indica* L. PLOS One 9, e114196 (2014). 25464336 10.1371/journal.pone.0114196 PMC4252105

[b55] Fu J. & Wang S. Insights into auxin signaling in plant-pathogen interactions. Front. Plant Sci. 2, 74 (2011). 22639609 10.3389/fpls.2011.00074 PMC3355572

[b56] Mutka A. M., Fawley S., Tsao T. & Kunkel B. N. Auxin promotes susceptibility to *Pseudomonas syringae* via a mechanism independent of suppression of salicylic acid-mediated defenses. Plant J. 74, 746–754 (2013). 23521356 10.1111/tpj.12157

[b57] Yoshida S. . Mannosylerythritol lipids secreted by phyllosphere yeast *Pseudozyma antarctica* is associated with its filamentous growth and propagation on plant surfaces. Appl. Microbiol. Biotechnol. 98, 6419–6429 (2014). 24706213 10.1007/s00253-014-5675-x

[b58] Morita T. . Production of a novel glycolipid biosurfactant, mannosylmannitol lipid, by *Pseudozyma parantarctica* and its interfacial properties. Appl. Microbiol. Biotechnol. 83, 1017–1025 (2009). 19296097 10.1007/s00253-009-1945-4

[b59] Konishi M. . Yeast extract stimulates production of glycolipid biosurfactants, mannosylerythritol lipids, by *Pseudozyma hubeiensis* SY62. J. Biosci. Bioeng. 111, 702–705 (2011). 21393057 10.1016/j.jbiosc.2011.02.004

[b60] Maldonado A. M. . A putative lipid transfer protein involved in systemic resistance signalling in *Arabidopsis*. Nature, 419, 399–403 (2002). 12353036 10.1038/nature00962

[b61] Shah J. Lipids, lipases, and lipid-modifying enzymes in plant disease resistance. Annu. Rev. Phytopathol 43, 229–260 (2005). 16078884 10.1146/annurev.phyto.43.040204.135951

[b62] Narusaka M. . Yeast cell wall extract induces disease resistance against bacterial and fungal pathogens in *Arabidopsis thaliana* and *Brassica* crop. PLOS One 10, e0115864 (2015). 25565273 10.1371/journal.pone.0115864 PMC4286235

[b63] Moon H. N., Lee G. Y., Yun H. S. & Kwon C. A. Non-proteinaceous yeast extract induces *Arabidopsis* defense responses independently of salicylic acid. J. Plant Biol. 58, 38–43 (2015).

[b64] Castresana J. Selection of conserved blocks from multiple alignments for their use in phylogenetic analysis. Mol. Biol. Evol. 17, 540–552 (2000). 10742046 10.1093/oxfordjournals.molbev.a026334

[b65] Hall T. A. BioEdit: a user-friendly biological sequence alignment editor and analysis program for Windows 95/98/NT In Nucleic Acids Symposium Series 41 (ed. Hall T. A.) 95–98 (Oxford University Press, 1999).

[b66] Swofford D. L.PAUP*: phylogenetic analysis using parsimony, version 4.0 b10 (2003)

[b67] Abarenkov K. . The UNITE database for molecular identification of fungi–recent updates and future perspectives. New Phytol 186, 281–285 (2010). 20409185 10.1111/j.1469-8137.2009.03160.x

[b68] Saitou N. & Nei M. The neighbor-joining method: a new method for reconstructing phylogenetic trees. Mol. Biol. Evol. 4, 406–425 (1987). 3447015 10.1093/oxfordjournals.molbev.a040454

[b69] Felsenstein J. Confidence limits on phylogenies: an approach using the bootstrap. Evolution 39, 783–791 (1985). 10.1111/j.1558-5646.1985.tb00420.x 28561359

[b70] Sukumaran J. & Holder M. T. SumTrees: summarization of split support on phylogenetic trees. Part of the DendroPy Phylogenetic Computation Library Version, 2 (http://pypi.python.org/pypi/DendroPy) (2008).

[b71] Huson D. H. . Dendroscope: An interactive viewer for large phylogenetic trees. BMC Bioinformatics 8, 460 (2007). 18034891 10.1186/1471-2105-8-460 PMC2216043

[b72] Kang S. H. . Two bacterial endophytes eliciting both plant growth promotion and plant defense on pepper (*Capsicum annuum* L.). J. Microbiol. Biotech. 17, 96–103 (2007). 18051359

[b73] Ryu C. M., Murphy J. F., Mysore K. S. & Kloepper J. W. Plant growth-promoting rhizobacteria systemically protect *Arabidopsis thaliana* against *Cucumber mosaic virus* by a salicylic acid and NPR1-independent and jasmonic acid-dependent signaling pathway. Plant J. 39, 381–392 (2004). 15255867 10.1111/j.1365-313X.2004.02142.x

[b74] Niu D. D. . The plant growth- promoting rhizobacterium *Bacillus cereus*AR156 induces systemic resistance in *Arabidopsis thaliana* by simultaneously activating salicylate- and jasmonate/ethylene-dependent signaling pathways. Mol. Plant Microbe Ineract. 24, 533–542 (2011). 10.1094/MPMI-09-10-0213 21198361

[b75] Hahm M. S. . Biological control and plant growth promoting capacity of rhizobacteria on pepper under greenhouse and field conditions. J. Microbiol. 50, 380–385 (2012). 22752900 10.1007/s12275-012-1477-y

[b76] De Beer E. J. & Sherwood M. B. The paper-disc agar plate method for the assay of antibiotic substances. J. Bacteriol. 50, 459–467 (1945). PMC374159 16561019

[b77] Heil M., Hilpert A., Kaiser W. & Linsenmair K. E. Reduced growth and seed set following chemical induction of pathogen defence: Does systemic acquired resistance (SAR) incur allocation costs? J. Ecol. 88, 645–654 (2000).

